# HIV and Substance Use Reduction for Youth Experiencing Homelessness: Development and Usability Study

**DOI:** 10.2196/82232

**Published:** 2026-05-14

**Authors:** Diane Santa Maria, Michael Businelle, Natasha Slesnick, Nikhil Padhye, Traquel Harrison, Luke Ging, Jennifer Diane Torres Jones, Margaret White

**Affiliations:** 1 Cizik School of Nursing, The University of Texas Health Science Center at Houston, 6901 Bertner Avenue, Houston, TX, 77030, United States, 1 713-500-2002; 2 TSET Health Promotion Research Center, University of Oklahoma Health Sciences Center, Oklahoma City, OK, United States; 3 Department of Human Sciences, College of Education and Human Ecology, The Ohio State University, Columbus, OH, United States

**Keywords:** youth experiencing homelessness, HIV prevention, substance use prevention, intervention development, just-in-time adaptive intervention

## Abstract

**Background:**

Youth experiencing homelessness face heightened vulnerability to HIV infection and substance use due to complex structural, psychosocial, and behavioral factors. Despite increased mobile phone access among youth experiencing homelessness, few mobile health interventions have been tailored to their unique needs, and even fewer have applied behavioral theory to inform message development.

**Objective:**

This study aimed to develop and refine theory-driven, tailored HIV prevention and substance use reduction messages for use in a just-in-time adaptive intervention app, MY-RIDE (Motivating Youth to Reduce Infections, Disconnections, and Emotional dysregulation), designed for youth experiencing homelessness aged 18 to 25 years.

**Methods:**

This study was conducted in 4 phases: prevention messages were developed and pilot-tested in 2018 (phase 1), revised and expanded using the experience and expertise of content experts and the study team (phase 2), reviewed for relevance and acceptability by youth experiencing homelessness in 2024 (phase 3), and supplemented with messages generated using an artificial intelligence (AI) tool (phase 4).

**Results:**

Phase 1 resulted in the development of 386 intervention messages across 7 content categories: sex urge, drug and alcohol urge, stress, drug use, recent sexual activity, recent sexual assault, and general motivational messages. During phase 2, the study team expanded the message library to 888 messages across 10 categories. During phase 3, the youth working group liked 93% (803/864) of messages reviewed, which were categorized as acceptable for the intervention. Disliked messages were discarded and replaced with messages generated by an AI tool in phase 4.

**Conclusions:**

The finalized set of intervention messages was integrated into the MY-RIDE app to support personalized, real-time intervention delivery. Codeveloping messages with youth experiencing homelessness and leveraging AI tools proved feasible and effective for tailoring HIV prevention and substance use content. This approach supports scalable mobile health interventions for marginalized populations and informs future efforts to design engaging, theory-based digital health strategies. A randomized controlled trial of the MY-RIDE intervention is underway.

## Introduction

Youth experiencing homelessness are disproportionately affected by a constellation of health challenges, including heightened rates of HIV infection, substance use, and exposure to chronic stress [1,2]. HIV prevalence among youth experiencing homelessness is significantly higher than among their stably housed peers, driven by complex factors such as limited access to health care, engagement in high-risk sexual behaviors, survival sex, and substance use [3,4]. Chronic stress resulting from unstable housing, trauma, and marginalization further compounds vulnerability to HIV, both by impairing cognitive function and by reducing the capacity to engage in preventive behaviors [5,6].

Substance use is both a coping mechanism for stress and a direct risk factor for HIV acquisition among youth experiencing homelessness [7,8]. Illicit drug use, particularly injection drug use and stimulant use, is strongly associated with increased HIV risk through needle sharing and reduced inhibition during sexual encounters [9]. Moreover, substance use complicates efforts to adhere to pre-exposure prophylaxis (PrEP) or postexposure prophylaxis and other HIV prevention strategies, underscoring the need for tailored interventions that are responsive to the realities of youth living without stable housing [10,11].

Mobile health (mHealth) interventions represent a promising avenue for reaching youth experiencing homelessness with HIV prevention resources. Despite experiencing housing instability, the ubiquity of mobile phone access among homeless youth presents an opportunity to deliver health information in a discreet, accessible, and engaging manner [12]. Recent literature highlights the effectiveness of mHealth tools in promoting HIV testing, PrEP uptake, and medication adherence across various populations, including adolescents and at-risk populations [13,14]. However, relatively few mHealth interventions have been developed specifically for youth experiencing homelessness, and even fewer have incorporated theoretically grounded, tailored messaging frameworks.

The information-motivation-behavioral skills (IMB) model provides a useful theoretical framework for designing health behavior interventions, including those delivered through mHealth platforms. Developed by Fisher and Fisher [15] and later refined, the IMB model posits that individuals must be well informed, motivated, and possess the necessary behavioral skills to enact and sustain health-promoting behaviors [16]. In the context of HIV prevention, this involves providing accurate information about transmission and prevention, fostering personal and social motivation to reduce risk behaviors, and enhancing self-efficacy and skills for the consistent use of prevention tools such as condoms or for PrEP or postexposure prophylaxis uptake and adherence.

Several recent studies have applied the IMB and other models to the development of mHealth interventions targeting youth. A recent systematic review found 34 studies that used behavioral change techniques and reported positive results, including condom use self-efficacy and condom use [17]. Additionally, Wilder et al [18] developed and evaluated a mobile AI-augmented app tailored to the needs and lived experiences of youth experiencing homelessness, incorporating multimedia content and interactive features that addressed IMB constructs. Several mHealth interventions have leveraged tailored messaging to improve HIV prevention outcomes, particularly among youth and other high-risk populations. Tailoring involves adapting content to the characteristics, behaviors, or preferences of users to enhance relevance, engagement, and behavioral impact [13,19-22]. Tailored messaging has been shown to increase the effectiveness of digital interventions by making health communication more personally meaningful, which is particularly important for marginalized and heterogeneous populations such as youth experiencing homelessness. Together, these studies demonstrate the feasibility and acceptability of theory-based, tailored mHealth interventions [23]; however, they also highlight the need for more rigorous, longitudinal evaluations to assess sustained behavior change and impact on HIV incidence. These studies collectively suggest that tailoring digital HIV prevention messages, especially when grounded in behavioral theory and codeveloped with the target population, can improve engagement, perceived relevance, and health outcomes. However, further research is needed to evaluate the long-term efficacy and scalability of such interventions among unstably housed populations.

In summary, youth experiencing homelessness face a unique confluence of HIV risk factors rooted in structural vulnerability, stress and coping responses, and substance use. mHealth interventions grounded in behavioral theory, such as the IMB model, offer a scalable and youth-centered approach to HIV prevention. Expanding this body of research and integrating tailored, contextually salient messaging are critical to enhancing the effectiveness of HIV prevention efforts, addressing the specific needs of youth experiencing homelessness, and ultimately reducing disparities in HIV incidence within this marginalized population. In this paper, we describe the process of developing and refining messages for MY-RIDE (Motivating Youth to Reduce Infections, Disconnections, and Emotional dysregulation), an HIV prevention and substance use just-in-time adaptive intervention (JITAI) tailored to youth experiencing homelessness aged 18 to 25 years at risk for HIV.

## Methods

### Study Setting

This study was conducted in an urban area of southeast Texas. All study procedures were approved by the institutional review board (IRB) before participant recruitment. Study participants were recruited in 2018 and again in 2024 from drop-in centers, social service providers, and a young adult emergency shelter that provides services for youth experiencing homelessness. The drop-in centers serve meals; provide housing assistance and other community services; and offer clothing, pantry items, and hygiene products on a weekly basis. In addition to basic amenities, the young adult emergency shelter provides short- and long-term housing for individuals aged 18 to 25 years, as well as case management, mental health counseling, job and life skills training, and access to an onsite adolescent medicine clinic.

### Sampling Design and Participants

Convenience sampling was used to recruit youth to participate in a youth working group (YWG) to review and revise HIV and substance use prevention messages for the MY-RIDE intervention. Youth were invited to participate if they were aged 18 to 25 years and were currently or had recently experienced homelessness. Staff from the shelter and drop-in centers advertised the opportunity to residents and clients at least 1 week prior to each YWG meeting. Study staff recruited onsite by making general announcements and describing the purpose of the YWG. Written consent was obtained from each participant who agreed to participate in the YWG. Once consented, interested individuals met with staff either in a group or individually to review the consent form and were informed that the YWG would meet monthly to work on message development and revision. Individuals were notified that each meeting would last 1 to 2 hours, be conducted onsite, and be audio-recorded.

### Procedures and Data Collection

#### Overview

This study is presented in 4 phases. Prevention messages that were developed and pilot-tested in 2018 [24,25] served as the foundation for message review for the YWG (phase 1). The original messages revised in this study (approximately 300 intervention and control messages in total) were developed using an iterative process that also used the experience and expertise of a YWG, content experts, and the study team (phase 2). In 2024, messages were then further refined and revised using a newly formed YWG and a substance use prevention expert (phase 3), and the process incorporated the use of an AI tool for additional message generation (phase 4).

#### Phase 1: 2018 Pilot Study

In the 2018 pilot study, the team used an intervention mapping approach [26] to ensure that the message content reflected the cognitive, behavioral, and environmental factors related to HIV prevention among youth experiencing homelessness [27,28]. The study team conducted a literature review, assessed resources, and developed messages guided by social cognitive theory [29] and known HIV behavior change communication strategies [30]. The team convened 5 meetings with the YWG to review the initial slate of messages, create new messages, and suggest linguistic translations to ensure the messages were motivating, relatable, and culturally sensitive to the needs of youth experiencing homelessness. In total, 28 individuals participated in 1 or more meetings of the YWG, and the meeting size for each session was 10 participants. Participants completed a brief demographic survey on paper. All YWG meetings were held onsite (at the shelter or drop-in centers) immediately after recruitment. Each meeting was audio-recorded, and an observer took field notes.

#### Phase 2: Expand Messages Using Expertise of the Study Team

The study team started with messages tested in the 2018 pilot study to expand upon and refine them prior to presenting them to the 2024 YWG. Collectively, the investigative team has extensive experience developing theory-driven prevention messages for youth experiencing homelessness and has conducted several JITAI studies focused on behavioral science, adolescent health, youth experiencing homelessness, substance use, health communication, and personalized message–based prevention interventions. The study team that interacted with the YWG to develop messages included 2 research nurses, 2 research assistants, a research coordinator with a Master of Public Health degree, and an HIV specialist nurse practitioner, all of whom have at least 5 years of experience working with the target population. The team also included 2 undergraduate students within the participants’ age range who were pursuing degrees in nursing and nutrition.

#### Phase 3: Review and Revise Prevention Messages With the 2024 YWG

In 2024, we held 1 meeting to orient the YWG to the study and review study procedures, followed by 4 YWG meetings to re-evaluate the salience and acceptability of the pilot-tested prevention messages and to assess the need to add additional topics. Two meetings were held at a drop-in center, and 2 meetings were held at a shelter. In total, 24 individuals participated in 1 or more meetings of YWG, and meeting sizes ranged from 3 to 15 participants. Each participant completed a short demographic survey using REDCap (Research Electronic Data Capture) hosted at the University of Texas Health Science Center [31,32], and group facilitators outlined the specific meeting objectives, which were set by the study team and revised with YWG input. Each meeting was audio-recorded, and an observer took comprehensive field notes. Each meeting started with an icebreaker and a general orientation to the agenda, as well as an explanation of how these messages would be received in the future (via an app tailored to individual responses). All messages were programmed into REDCap, which allowed participants to individually rate the messages on an iPad using a 4-point Likert scale: 1=“Like a lot,” 2=“Like a little,” 3=“Dislike a little,” and 4=“Dislike a lot.” Every message was rated by at least 2 members of the YWG, and messages with an average rating of ≤2 (ie, liked a lot or a little) were retained. Messages rated 3 to 4 (ie, disliked a little or a lot) were reviewed for patterns and ultimately discarded. Messages with mixed ratings of 2.1 to 2.9 (liked and disliked) were rerated by a different member of the YWG to determine whether to retain or discard the message. Time for discussion was included in each meeting to gather additional feedback on specific messages. Participants provided feedback on the salience and acceptability of the messages and described their understanding of the message to determine if their interpretation matched the message’s intent. Messages were then rank-ordered within content categories to identify the most liked messages.

#### Phase 4: Use an AI Tool to Generate Additional Messages

The most highly rated messages, which scored an average of 1 (liked a lot), were extrapolated and used to generate additional, similar message content modeled after prompts from another study [33] using the free, web-based Microsoft Copilot (version GPT-4 Turbo). At the time of the YWG meetings, Microsoft Copilot was the only AI tool approved for use in research at the university. The AI prompt used was: *“*Create (number of messages needed) messages similar to these about (topic):” followed by 4 highly rated messages with at least 1 information-based, 1 motivation-based, and 1 behavior-based message.

For example, “Create 20 messages about stress similar to these:”

- “Do things seem overwhelming today? Listen to music to calm your nerves.”
- “Going for a walk can help relieve stress.”
- “Sharing bad experiences can help. Reach out to close friends or a relative.”
- “Decreasing stress can help improve your mood, increase resilience, and enhance overall mental well-being.”

### Data Analysis

Demographic survey responses were exported from REDCap, and descriptive statistics (frequencies with percentages and means with SDs) were generated using Microsoft Excel (version 2408 Build 16.0.17932.20742).

### Ethical Considerations

This study was reviewed and approved by the IRB of the University of Texas Health Science Center (HSC-SN-23-0360). Participant confidentiality was rigorously protected through procedures that adhered to the Declaration of Helsinki, as well as applicable national and institutional regulations. All participants provided written informed consent. The IRB waived the requirement of parental consent for unaccompanied minors because they met the federal criteria for a waiver of parental permission when such requirements are not reasonable or feasible. YWG participants received a US $20 gift card for each meeting as compensation for their time.

### Results

**Figure 1.**
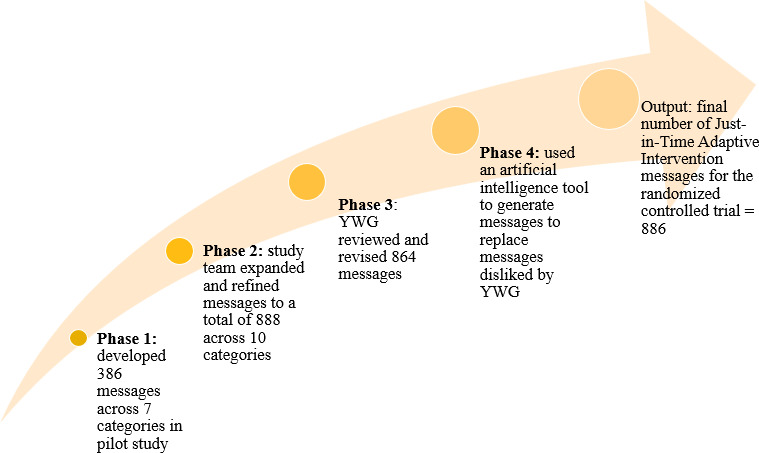
MY-RIDE (Motivating Youth to Reduce Infections, Disconnections, and Emotional dysregulation) message development process. YWG: youth working group.

The results from each step of the message development process are summarized in Figure 1.

#### Phase 1: 2018 Pilot Study

The pilot study team developed 187 original intervention messages in 2018 after an extensive literature review and assessment of existing resources related to HIV prevention and substance use, including websites, social media accounts, and educational materials from local and national organizations. The pilot YWG met 5 times to rate and revise the messages developed by the study team and to write new messages that were relevant and acceptable. Demographic characteristics for the 2018 YWG are provided in Table 1.

**Table 1.** Demographic characteristics of the 2018 and 2024 youth working groups (YWGs).

Characteristic	2018 YWG (n=28), n (%)	2024 YWG (n=24), n (%)
Age (years), mean (SD)	21.7 (1.68)	22.0 (2.39)
Race or ethnicity		
American Indian or Alaska Native	0 (0)	1 (4.1)
Black	18 (64.3)	11 (45.8)
Hispanic	3 (10.7)	4 (16.7)
Multiple races	3 (10.7)	4 (16.7)
White	4 (14.3)	4 (16.7)
Gender		
Man	15 (53.6)	11 (45.8)
Woman	12 (42.8)	12 (50.0)
Nonbinary	1 (3.6)	1 (4.2)
Sexual orientation		
Heterosexual	15 (53.6)	16 (66.7)
Bisexual	3 (10.7)	4 (16.6)
Gay	4 (14.3)	1 (4.2)
Pansexual	4 (14.3)	1 (4.2)
Lesbian	2 (7.1)	2 (8.3)

Youth expressed that some risk reduction messages, especially those related to drug use, were perceived as endorsing the behavior and often recommended risk avoidance alternatives. For example, messages such as “Before you drink or use drugs, be sure you are in a safe place” or “If you inject drugs, use only new, sterile needles each time you inject” yielded alternatives such as “Don’t do drugs” and “Injecting drugs isn’t safe.” This sentiment was also expressed in sexual contexts, with 1 participant sharing, “I say you shouldn’t have sex before marriage. That would eliminate a lot of this.”

Others endorsed drugs and/or alcohol, particularly marijuana, writing that “marijuana is inspirational” and “sometimes weed is good for you.” One participant proposed replacing the message “You seem stressed right now. Call and talk to someone” with “Smoke a blunt.”

A key takeaway was that motivational messages were important to this group, and concise messages were preferred. One participant described the message “Repeat this to yourself: ‘I am strong, I can get through this without using alcohol’” as an “outstanding message.” Examples of message ideas included “Strap it up,” “Better safe than sorry,” “Be careful who you surround yourself with,” “Think before you act,” “Better days are to come!” and “Down with dope and up with hope.”

After compiling the information gathered from the YWG, 386 intervention messages were developed across 7 content categories (Table 2): sex urge (n=94, 24.3%), drug and alcohol urge (n=100, 25.9%), stress (n=27, 7%), drug use (n=37, 9.6%), recent sexual activity (n=51, 13.2%), recent sexual assault (n=8, 2.1%), and general motivational messages that would be provided when no risk is detected in the JITAI (n=69, 17.9%). These messages were pilot-tested and found to reduce drug use and sexual urge in the intervention group and to reduce stress in both groups [24].

**Table 2.** Number of intervention messages categorized by topic throughout the development process.

Topic	Messages from the 2018 pilot (n=386), n (%)	Messages after the 2024 study team expansion (n=888), n (%)	Messages after 2024 youth working group (n=886), n (%)
Sex urge	94 (24.3)	100 (11.3)	100 (11.3)
Drug urge	100 (25.9)	103 (11.6)	99 (11.2)
Alcohol urge	0^a^ (0)	141 (15.9)	137 (15.5)
Stress	27 (7.0)	109 (12.3)	107 (12.1)
Recent drug use	37 (9.6)	101 (11.4)	100 (11.3)
Injected drugs^b^	0^a^ (0)	10 (1.1)	30 (3.4)
Recent sexual activity	51 (13.2)	100 (11.3)	100 (11.3)
Used alcohol	0^a^ (0)	106 (11.9)	99 (11.2)
Sexual assault^b^	8 (2.1)	14 (1.6)	14 (1.6)
General motivation	69 (17.9)	104 (11.7)	100 (11.3)

a In the pilot study, drug and alcohol messages were in the same category, and messages related to needle use were in the same category as used drugs.

b Not tested by the youth working group.

#### Phase 2: Expansion of Messages Using the Expertise of the Study Team

Messages were then divided among the study team, who removed outdated local resources or language and segmented broader categories into smaller groups to respond to recent behaviors or currently occurring urges to use drugs, consume alcohol, or have sex, which are antecedents of HIV risk. The study team rotated messages across categories twice to review, edit, and create additional messages. Finally, the investigative team reviewed, edited, and suggested additional categories not covered in the original messages but that were endorsed by youth experiencing homelessness, such as injection drug use. This process resulted in a total of 888 messages across 10 categories (Table 1): sex urge (n=100, 11.3%), drug urge (n=103, 11.6%), alcohol urge (n=141, 15.9%), stress (n=109, 12.3%), recent drug use (n=101, 11.4%), injected drugs (n=10, 1.1%), recent sexual activity (n=100, 11.3%), used alcohol (n=106, 11.9%), recent sexual assault (n=14, 1.6%), and motivational messages provided when no urge or risk is detected (n=104, 11.7%).

#### Phase 3: Review and Revision of Prevention Messages With the 2024 YWG

Four YWG meetings were held to specifically gather feedback on the messages and assess their likability. Demographic characteristics for the 2024 YWG are provided in Table 1. Due to the low frequency of injection drug use among youth experiencing homelessness in the region across several past studies [24] and the sensitive nature of sexual assault, messages in these 2 categories were not reviewed by the YWG (but were reviewed by the study team). Demographic characteristics for the YWG are provided in Table 1. To work within the study timeline, the study team focused on having the YWG score messages, with the goal of eliminating the least liked messages.

The YWG reviewed 864 messages across 3 meetings. Of these, 231 (26.7%) messages with scores of 2.1 to 2.9 (liked and disliked) were rerated by another member of the YWG to determine whether to retain or discard the message. Ultimately, 93% (n=804) of messages had an average score of ≤2 (liked a lot or a little) and were categorized as acceptable for the intervention. The YWG disliked 7% (n=60) of messages, which were discarded.

#### Phase 4: Using an AI Tool to Generate Additional Messages

The highest-rated messages in each category, scoring 1 (liked a lot by all YWG raters), were reviewed, and 4 sample messages per category were chosen to prompt the AI tool, Copilot.

Table 3 lists all AI prompts. The output (n=146) was reviewed for redundancy, and 70 (47.9%) messages were chosen for testing among the YWG. The low-scoring (“disliked”) messages were replaced with the 70 messages generated by Copilot, which were tested in a fourth YWG meeting for understandability and salience. All 70 messages were liked by the majority of the participants (n=69, 99%), with a mean score of 1.29 (SD 0.33).

**Table 3.** Artificial intelligence prompts.

Topic	Prompt
Sex urge	“Create 20 messages about sex urge similar to these: Stay healthy, only have sex with condoms. Condoms offer a barrier against STIs^a^ and pregnancy. No glove, no love. Trust your gut and know when it’s best to wait for the right moment to have sex.”
Drug urge	“Create 30 messages about drug urge similar to these: Drugs will never improve your situation. Choose yourself. Put yourself first, say no to getting high. Your drug urge is high. You have the right to speak up for yourself, say no. Your craving for drugs is strong. Resist the temptation and walk away.”
Stress	“Create 20 messages about stress similar to these: Do things seem overwhelming today? Listen to music to calm your nerves. Going for a walk can help relieve stress. Sharing bad experiences can help. Reach out to close friends or a relative. Decreasing stress can help improve your mood, increase resilience, and enhance overall mental well-being.”
Drug use	“Create 12 messages about using drugs similar to these: Remember if you’re going to be high, don’t have sex. It can make using a condom more difficult. Let someone know where you are before using in case you need help out of a dangerous situation. Peer pressure is real. Make decisions that align with your values, not other people’s expectations. Find healthy coping mechanisms for stress or difficult emotions.”
Injection drug use	“Create 40 messages similar to these about drug needle use: Don’t inject drugs. If you do, be sure to use a clean needle. Did you know you can get a disease from sharing needles? The tiniest drop of blood left inside a needle has the power to infect another person. Don’t share needles, it can infect you and others. Always use a clean needle.”
Had sex	“Create 14 messages about having sex similar to: PrEP^b^ is a pill you take once a day that can prevent you from getting HIV. If you test HIV positive, there are meds you can take to help you live a long and healthy life, and reduce the risk of giving HIV to someone else. Had condomless sex, no need to keep worrying visit one of the many clinics and get a free HIV & STI exam. Unprotected sex can lead to anxiety, stress, and emotional distress, particularly if there are concerns about STIs & HIV.”
Motivational messages	“Create 10 messages similar to these: Be proud of how far you’ve come, and have faith in how far you can go. Believe in your ability to create a beautiful life. What’s your goal for today? Pick one thing to help you get where you want to go. It may be hard to get to where you want to go, but not impossible. Get a little closer each day.”

a STI: sexually transmitted disease.

b PrEP : pre-exposure prophylaxis.

Overall, the YWG rated 934 messages: 864 (92.5%) messages from the 2018 pilot and those generated by the study team in 2024, and 70 (7.5%) messages generated by Copilot. In total, 886 (94.9%) messages were programmed into the Insight mHealth Platform (University of Oklahoma Health Sciences Center) to test the efficacy of MY-RIDE on HIV prevention and substance use reduction (Table 2). Messages were programmed so that the highest-scoring, or most liked, messages were delivered first, with users receiving alternating message types (information-, behavioral-, or motivation-oriented).

## Discussion

### Principal Results

This study describes the process used to codevelop relevant and acceptable HIV prevention and substance use reduction messages used for the JITAI app, MY-RIDE. The primary goal was to increase the number of messages developed during the 2018 pilot and to ensure their acceptability for the target population.

During phase 1, the YWG sessions held in 2018 resulted in important considerations for the team, namely, a preference for concise wording and an affinity for motivational messages. These messages were pilot-tested and found to reduce drug use and sexual urge in the intervention group and to reduce stress in both groups [24]. Phase 2 represented a key step in building on this foundation with promising results and expanding the intervention to achieve the volume of messages needed for a randomized controlled trial. As researchers are often tasked with building and testing interventions over the course of several years, this is a critical step in balancing the integrity of foundational work with necessary updates to reflect new language, trends, and resources. In phases 3 and 4, the 2024 YWG liked the vast majority of messages (>90%), demonstrating that using a multiphase approach that leverages results from a pilot study, incorporates expertise from the research team, gathers feedback from YWGs, and uses the power of AI tools is a promising avenue to ensure acceptability while using limited resources. It is also notable that AI-generated messages were well liked, which can save substantial time in message generation for mHealth interventions [33].

Acceptable messages were programmed into the study app developed by MB at the University of Oklahoma Health Sciences Center, a coinvestigator on this study. He has expertise in smartphone-based interventions focused on cigarette cessation [34], alcohol reduction or cessation [35], and physical inactivity [36], and he supported the programming of study measures and messaging. The research team then conducted beta testing to confirm app functionality and correct any issues in preparation for a fully powered randomized controlled trial of MY-RIDE [37]. To our knowledge, an app like this has not previously been tested for HIV and substance use among youth experiencing homelessness.

To address the HIV epidemic among youth experiencing homelessness, interventions must go beyond access to care and awareness building [38]. The tumultuous living circumstances and histories of trauma often experienced by youth experiencing homelessness warrant a different approach that can be addressed via JITAIs. Intervention messages can be programmed to be automatically delivered to youth experiencing homelessness smartphones during times of need and to respond to state-level antecedents of HIV risk and substance use behaviors [39-41]. Furthermore, integrated, context-sensitive intervention messages must address both substance use and mental health symptoms [42]. The high ratings of the current intervention messages suggest that our iterative and rigorous message development process was effective in creating salient messages that address the challenges youth experiencing homelessness face in their HIV and substance use prevention efforts. Furthermore, the messages were developed over time and reflect both pre– and post–COVID-19 pandemic acceptability. Although this process was used specifically for topics related to HIV and substance use, it may be applicable to other health and housing challenges faced by youth experiencing homelessness and should be more widely explored.

### Limitations

Although the message development and evaluation process was theory driven and participatory, it is not without limitations. First, the YWGs consisted of a convenience sample of youth experiencing homelessness. Although the sample was diverse in terms of gender and race or ethnicity, it may not have represented all youth experiencing homelessness in the area.

Second, the team did not beta test all messages due to the volume of messages and the sensitive nature of the sexual assault topics. Although the injection drug use and sexual assault messages were not generally tested with the YWG, they were carefully examined by the experienced study team. Third, the messages were reviewed outside the context of the study; therefore, they may not have been applicable to the YWG member rating the message (eg, a non–drug user scoring a message regarding drug use). We were unable to replicate the experience of receiving a message tailored to an individual without asking personal questions; however, group facilitators spent substantial time in each meeting describing how YWG members should score messages, assuming relevance to the person receiving the message. Finally, the intervention development did not originally include a plan to incorporate messages generated by AI. Consequently, we did not test these messages to compare their likeability with that of messages written by the YWG or the study team. However, given the availability of this new technology and the timing of the study, the team quickly integrated generative AI strategies to test their applicability.

### Conclusions

Using a YWG and AI to maximize the development of intervention messages was a feasible way to increase and beta test intervention messages before launching a randomized trial. The expansion of mHealth prevention strategies requires leveraging tools to maximize human and financial resources, particularly in highly transient populations that face additional barriers to accessing traditional multisession, in-person interventions. A randomized attention control trial of MY-RIDE is currently underway [37].

## Abbreviations

IMB: information-motivation-behavioral skills
IRB: institutional review board
JITAI: just-in-time adaptive intervention
mHealth: mobile health
MY-RIDE: Motivating Youth to Reduce Infections, Disconnections, and Emotional dysregulation
PrEP: pre-exposure prophylaxis
REDCap: Research Electronic Data Capture
YWG: youth working group
